# Partitioning of respiratory mechanics after cardiac arrest and cardiopulmonary resuscitation

**DOI:** 10.1186/2197-425X-3-S1-A271

**Published:** 2015-10-01

**Authors:** F Ruiz Ferron, JM Serrano Simón

**Affiliations:** Complejo Hospitalario de Jaen, Intensive Care Unit, Jaen, Spain; Hospital Universitario Reina Sofía, Intensive Care Unit, Córdoba, Spain

## Introduction

Acute respiratory failure is a frequently complication after cardiac arrest. These patients need mechanical ventilation, however there is a scare information about the components of respiratory mechanics.

## Objectives

To evaluate the total respiratory system mechanics into the lung and chest wall mechanics using the esophageal balloon technique after chest compressions and artificial ventilation post-cardiac arrest.

## Methods

The study was conducted in general intensive care of two tertiary hospitals. 15 patients were studied after hypothermia in clinically stable situation. During volume assist control mechanical ventilation, with constant flow. Esophageal pressure were measured using balloon catheter, positioned by gastric compression procedure. Airways pressure, respiratory flow and esophageal pressure were registered at 100 Hz, and posterior analysis was performed by Anadat® software. The passive respiratory mechanics analysis were measured without external PEEP and the other respiratory parameters of mechanical ventilation were unchanged. Multiple linear regression techniques were used to calculate respiratory mechanics and his components, without interruption of mechanical ventilation. Data are expressed as mean ± standard deviation and range.

## Results

Ventilator parameters were: Vt 0,6 ± 0,1, V´I:0,64 ± 0,1L/s, TI:1,0 ± 0,3 s, Ttot:2,4 ± 0,5 s, RR:17 ± 4 bpm, Fi02:0,6 ± 0,2, PEEPe: 0 cmH2O. Respiratory system mechanics: Ers 34 ± 12 cmH2O/l, Rrs 15 ± 5 cmH2O/l/s, PEEPi 5 ± 0.7 cmH2O. Chest wall mechanics were, Ecw:10 ± 4 cmH2O/l, Rcw:0.7 ± 0.4 cmH2O/l/s. End esophageal espiratory pressure was 15 ± 5 (12-19 cmH2O). The mean fraction of Ecw to Ers was 32 ± 18 % (4 to 82 %).

## Conclusions

Total respiratory system mechanics were abnormal after of chest compressions post-cardiac arrest, a reflection of alteration of lung mechanics. Chest wall compliance showed high variability, similar to other critically ill patients. Higher end espiratory esophageal pressure must be considered in presence of severe respiratory failure.

